# Thanks to our peer reviewers

**DOI:** 10.1002/lrh2.10397

**Published:** 2023-10-17

The publication of Issue 4 marks the completion of Volume 7 of *Learning Health Systems*. An international, trans‐disciplinary, open access publication, the journal has advanced research and scholarship on learning health systems in partnership with our reviewers. With indexing in multiple major sources and the recent news that we have received our first official Impact Factor, we have achieved a publication milestone that signals a sustainable, positive trajectory. Articles from the journal were downloaded over 109,532 times in 2022.

The journal has now published seven *Special Issues*: “Patient Empowerment and the Learning Health System” (v.1); “Ethical, Legal, and Social Implications of Learning Health Systems” (v.2); “Learning Health Systems: Connecting Research to Practice Worldwide” (v.3); “Human Phenomics and the Learning Health System” (v.4); “Collaborative Learning Health Systems: Science and Practice” (v.5); and “Education To Meet the Multidisciplinary Workforce Needs of Learning Health Systems” (v.6). “Transforming Health Through Computable Biomedical Knowledge (CBK)” (v.7). Our talented guest editors have been instrumental in helping these *Special Issues* come to fruition.

We are keenly aware that these achievements would not have happened without the dedicated efforts and insightful comments of all those individuals who accepted invitations to review submitted articles. With busy schedules and full commitments, these individuals found the time and energy to contribute their expertise to our authors to help ensure that their papers met (and often exceeded) the journal's high standards for publication.

Please accept our sincere gratitude for your outstanding efforts.

*Charles P. Friedman*, Editor in Chief.

***Learning Health Systems* Peer Reviewers**

*Note*: These are the Reviewers for articles *published* in all four issues of Volume 7 and for all articles currently *published* and *posted online* in Early View.

Julia Adler‐Milstein (United States)

Tesfa Michael Alaro (Ethiopia)

Eta Berner (United States)

Sarah Birken (United States)

Juli Bollinger (United States)

Nicholas Bowersox (United States)

Erica Breuer (Australia)

Melinda Buntin (United States)

Michael Cantor (United States)

Jonathan Casey (United States)

Harold Collard (United States)

Marisa Conte (United States)

Derek Corrigan (Ireland)

Catherine Diederich (United States)

Margo Edmunds (United States)

Ayca Erdogan (United States)

Amanuel Ergado (Ethiopia)

Stephan Fihn (United States)

Allen Flynn (United States)

Thomas Foley (United Kingdom of Great Britain and Northern Ireland)

Tina Foster (United States)

Brandy Fureman (United States)

Sarah Greene (United States)

Robert Greenes (United States)

Gary Groot (Canada)

W. Ed Hammond (United States)

Kevin Haynes (United States)

Peter Kaboli (United States)

Dipak Kalra (Belgium)

Amy Kilbourne (United States)

Martin Kohn (United States)

Greg Koski (United States)

Yong‐Hong Kuo (Hong Kong)

Alison Laycock (Australia)

Xiaojuan Li (United States)

Nancy Lorenzi (United States)

Paula Lozano (United States)

David McCallie (United States)

Jamie McCusker (United States)

Mark McGilchrist (United Kingdom of Great Britain and Northern Ireland)

Scott Mahoney (South Africa)

Brad Malin (United States)

Teri Manolio (United States)

Brian Martin (United States)

Graham Martin (United Kingdom of Great Britain and Northern Ireland)

Blackford Middleton (United States)

Stephanie Morain (United States)

Cheryl Moyer (United States)

Mark Musen (United States)

Brian Ostasiewski (United States)

Gretchen Piatt (United States)

Jodyn Platt (United States)

Leon Pretorius (South Africa)

Andrew Read (United States)

Rachel Richesson (United States)

Frank Rockhold (United States)

Joshua Rubin (United States)

Sameer Saini (United States)

Lucy Savitz (United States)

Philip Scott (United Kingdom of Great Britain and Northern Ireland)

Jean Soler (Malta)

Matthew South (United Kingdom of Great Britain and Northern Ireland)

Jessie Tenenbaum (United States)

Alexandra Vinson (United States)

Shyam Visweswaran (United States)

Anita Walden (United States)

Jim Walker (United States)

Mark Weiner (United States)

Berta Whitney (Canada)

Jun Yasuda (Japan)

Yili Zhang (United States)

